# Flexible genomic island conservation across freshwater and marine *Methylophilaceae*

**DOI:** 10.1093/ismejo/wrad036

**Published:** 2024-01-10

**Authors:** Paul Layoun, Mario López-Pérez, Jose M Haro-Moreno, Markus Haber, J Cameron Thrash, Michael W Henson, Vinicius Silva Kavagutti, Rohit Ghai, Michaela M Salcher

**Affiliations:** Department of Aquatic Microbial Ecology, Institute of Hydrobiology, Biology Centre CAS, 37005 Ceske Budejovice, Czech Republic; Faculty of Science, University of South Bohemia, 37005 Ceske Budejovice, Czech Republic; Evolutionary Genomics Group, División de Microbiología, Universidad Miguel Hernández, 03550 San Juan de Alicante, Spain; Evolutionary Genomics Group, División de Microbiología, Universidad Miguel Hernández, 03550 San Juan de Alicante, Spain; Department of Aquatic Microbial Ecology, Institute of Hydrobiology, Biology Centre CAS, 37005 Ceske Budejovice, Czech Republic; Department of Biological Sciences, University of Southern California, Los Angeles, CA 90089, USA; Department of Geophysical Sciences, University of Chicago, Chicago, IL 60637, USA; Department of Aquatic Microbial Ecology, Institute of Hydrobiology, Biology Centre CAS, 37005 Ceske Budejovice, Czech Republic; Faculty of Science, University of South Bohemia, 37005 Ceske Budejovice, Czech Republic; Department of Aquatic Microbial Ecology, Institute of Hydrobiology, Biology Centre CAS, 37005 Ceske Budejovice, Czech Republic; Department of Aquatic Microbial Ecology, Institute of Hydrobiology, Biology Centre CAS, 37005 Ceske Budejovice, Czech Republic

**Keywords:** genomic microdiversity, genomics, genome-streamlined bacteria, Methylophilaceae, genomic islands, cultivation

## Abstract

The evolutionary trajectory of *Methylophilaceae* includes habitat transitions from freshwater sediments to freshwater and marine pelagial that resulted in genome reduction (genome-streamlining) of the pelagic taxa. However, the extent of genetic similarities in the genomic structure and microdiversity of the two genome-streamlined pelagic lineages (freshwater “*Ca.* Methylopumilus” and the marine OM43 lineage) has so far never been compared. Here, we analyzed complete genomes of 91 “*Ca.* Methylopumilus” strains isolated from 14 lakes in Central Europe and 12 coastal marine OM43 strains. The two lineages showed a remarkable niche differentiation with clear species-specific differences in habitat preference and seasonal distribution. On the other hand, we observed a synteny preservation in their genomes by having similar locations and types of flexible genomic islands (fGIs). Three main fGIs were identified: a replacement fGI acting as phage defense, an additive fGI harboring metabolic and resistance-related functions, and a tycheposon containing nitrogen-, thiamine-, and heme-related functions. The fGIs differed in relative abundances in metagenomic datasets suggesting different levels of variability ranging from strain-specific to population-level adaptations. Moreover, variations in one gene seemed to be responsible for different growth at low substrate concentrations and a potential biogeographic separation within one species. Our study provides a first insight into genomic microdiversity of closely related taxa within the family *Methylophilaceae* and revealed remarkably similar dynamics involving mobile genetic elements and recombination between freshwater and marine family members.

## Introduction

Marine and freshwater pelagic habitats are numerically dominated by small, free-living microbes with reduced genomes (<1.5 Mbp), mostly referred to as genome-streamlined [1]. These microbes have highly reduced genomes with few pseudogenes and paralogs, short intergenic spacers, and a low-genomic G + C content to enable an energy-conserving lifestyle suited to their oligotrophic habitats [1]. Examples include marine *Prochlorococcus* [2, 3], “*Ca.* Pelagibacterales” (marine SAR11; [4, 5]; freshwater LD12 [6]), *Methylophilaceae* (freshwater “*Ca.* Methylopumilus” [7]; marine OM43 [8]), and *Actinobacteriota* (marine “*Ca.* Actinomarina” [9, 10]; freshwater “*Ca.* Nanopelagicales” [11, 12], *Rhodoluna* and *Aquiluna* [13, 14])*.*

A streamlined lifestyle comes at the cost of reduced genomic flexibility. However, enough genomic variability necessary to avoid population collapse due to phage infections and competition over resources has to be maintained [15]. Most such genetic differences are localized in genomic regions called “flexible genomic islands” (fGIs) [16]. fGIs are frequently subject to gene gain by horizontal gene transfer and gene loss. Two distinct types of fGIs have been proposed as a consequence of foreign DNA uptake dynamics, replacement fGIs, and additive fGIs. Replacement fGIs are the result of homologous recombination and show little sequence similarity among each other despite similar functional roles. They often contain genes involved in glycocalyx-modifying mechanisms that aid in phage predation avoidance [16]. Additive fGIs are the product of insertions mediated by mobile genetic elements such as transposons, integrons, and phages that occur often at the same integration sites leading to the extension of the island [16]. Examples of such genomic microdiversity are *Prochlorococcus* ecotypes with adaptations to different light regimes [3, 17], “*Ca.* Nanopelagicales” strains with diversified carbohydrate transporters [12], and SAR11 strains with differences in phosphorus and purine metabolism [5]. A variability in genes involved in phage defense was also common in these examples [3, 5, 12, 17]. Thus, analyzing the microdiversity of streamlined organisms proves paramount to understand their adaptations to variable environmental factors and their ecology at large.

In this work, we analyzed the *Gammaproteobacteria* family *Methylophilaceae*, which includes closely related streamlined freshwater (LD28 clade, “*Ca.* Methylopumilus” [7]) and marine (OM43 [8]) methylotrophs. These taxa are widespread in aquatic environments constituting up to 4% of the bacterioplankton in freshwater lakes [7, 18] and surface seawater [19, 20]. Habitat transitions from freshwater sediments to freshwater pelagial and further to the marine realm have been proposed in the evolutionary history of this family [21]. While the transition from sediment to the pelagial of lakes was characterized by substantial genome reduction, the transition from freshwater to marine habitats was accompanied by adaptations to higher salinity through the acquisition of osmoregulation genes [21] and extensive proteome remodeling [22, 23]. Both streamlined freshwater and marine lineages share identical methylotrophic pathways [21], and their genomic microdiversity, however, remains so far unexplored. Hence, we analyzed the genomic structure, diversity, and the genetic repertoire of 103 complete genomes of “*Ca.* Methylopumilus” and OM43 obtained from cultures to identify conserved patterns spanning the family.

## Material and Methods

### Sampling and isolation of strains

Novel strains of “*Ca.* Methylopumilus spp.” were isolated from the epilimnion and hypolimnion of 14 lakes of varying size, depth, and trophic state in Central Europe (Table S1). All lakes were sampled during spring and autumn 2019, and six lakes were additionally sampled during summer 2019. Five milliliter samples from each sampling depth were filtered through 0.4-μm filters, 1:1 diluted with a C1-rich medium [21], and incubated at 16°C for 24–48 hours to select for methylotrophs. Thereafter, dilution to extinction with approximately 1 cell per well was conducted in 96-well-plates containing 1.5 ml of medium [24]. Growth was checked after 6–8 weeks of incubation at 16°C using a Cytoflex flow cytometer (Beckman Coulter; Brea, CA, USA) equipped with a blue (488 nm) laser (bandpass filters 525/40 and 690/50) after staining of a 190 μl subsample with SYBRGreen I (0.5× standardized concentration, Lonza, Rockland, ME, USA). All wells with microbial growth were transferred to fresh medium and further maintained by 1:10 transfers every 6–8 weeks, and glycerol stocks and 16S rRNA gene screening and analyses were performed as previously described [7, 12, 21]. Nine novel OM43 strains were isolated from coastal samples from the Gulf of Mexico between 2014–2017 [25, 26].

Filters for metagenomic analysis were collected during each lake sampling. For the Římov Reservoir (Czech Republic), metagenomic samples were taken every 3 weeks during the ice-free period in 2018–2019 (Table S1). Water from both epi- and hypolimnion of each lake was prefiltered through a 20-μm mesh to remove larger organisms and debris, and sequentially filtered through 5-μm (STERLITECH PES membrane filters, USA) and 0.22-μm (Millipore express PLUS, Germany) polysulfone filters until clogged (1–11 l depending upon the lake). Filters were stored at −80°C until DNA extraction. Subsamples of the 0.22 μm filtered water were taken for nutrient (phosphorus, nitrogen, ions, and dissolved organic carbon) analyses [27]. A submersible multiparametric probe (YSI EXO2, Yellow Springs Instruments, Yellow Springs, OH, USA) was deployed to measure profiles of temperature, pH, chlorophyll *a*, and conductivity. Additionally, a multiwavelength submersible fluorescence probe (FluoroProbe; bbe-Moldaenke, Kiel, Germany) was deployed in the Římov Reservoir to differentiate distinct phytoplankton groups.

### Genome sequencing, assembly, and annotation

Thirty “*Ca.* Methylopumilus sp.” and 7 *Methylotenera* sp*.* (used as outgroup) strains isolated in 2019 and 22 “*Ca.* Methylopumilus sp.” isolated earlier [7, 21] were grown in 400 ml medium until late stationary phase. Cells were harvested by centrifugation at 14000 rpm for 60 minutes, and DNA was isolated using the MagAttract HMW DNA kit (Qiagen, Venlo, NL). Nine OM43 strains were grown, harvested by filtration on 0.2-μm filters, and DNA was extracted with the GenElute kit (Sigma Aldrich, St. Louis, MO, USA) or by phenol–chloroform extraction as previously described [6, 25, 26]. Pair end libraries (PE150) were sequenced on a NovaSeq 6000 instrument (Illumina; NOVOGENE, Hong Kong). Raw reads were trimmed using BBMap v36.x (https://github.com/BioInfoTools/BBMap/) and assembled with SPAdes v3.12.0 (using kmers 29,49,59,69,79,89,99,109,119,127) [28]. Most assemblies resulted in 1 or 2 contigs that could manually be curated to a circular chromosome after repeated rounds of mapping of trimmed reads to contigs with Geneious 10 (default mapper, high sensitivity; www.geneious.com), extending contigs on both ends, identifying overlapping ends, and assembling with the Geneious 10 assembler (*de novo* assembly, high sensitivity). In a few cases without overlapping ends, we closed the genome by designing primers bordering gaps, PCR amplification, and Sanger sequencing of the amplicon (Table S2). Finally, 51 “*Ca.* Methylopumilus sp.”, 7 “*Methylotenera* sp.”, and 9 OM43 genomes were assembled and closed for this study (Table S3). One genome (RI-55) remained in one contig but was not circular. Published genomes of “*Ca.* Methylopumilus sp.” (*n* = 39, [21]) and of the OM43 clade (*n* = 3, [29, 30]) were included for further analysis. PROKKA [31] was used for gene prediction, and annotation was done with hmmsearch [32] against COG [33], TIGRFAM [34], and KEGG databases [35, 36].

### Comparative genomic analyses

All 110 genomes were used to compute average nucleotide (ANI) and average amino acid identities (AAI) to delineate species using a 95% identity threshold [37, 38]. The pangenome of each species was computed by all-vs.-all comparisons of all proteins for each genome using BLASTp (≥ 50% identity and ≥ 50% coverage cutoffs to define an ortholog). Phylogenomic trees were constructed by first using hmmsearch [32] to find TIGRFAMs shared among most genomes to create a list of 851 marker proteins (Table S4). All genomes included at least 55% (468) of these markers that were aligned using PRANK v.150803 (−protein +F) [39], trimmed using BMGE (−m BLOSUM30 -t AA -g 0.5 -b 3) [40], and concatenated (https://github.com/nylander/catfasta2phyml). A maximum likelihood tree was constructed with the 7 *Methylotenera* strains as outgroup using IQ-TREE (−bb 1000, −alrt 1000) [41] with ultrafast bootstrapping and the model WAG+F+R10, chosen by ModelFinder [42].

To further assess the genomic microdiversity, parsnp [43] was used to identify single nucleotide polymorphisms (SNPs) and the R package orthologr [44] to provide large-scale comparative dN/dS estimations based on a reference (references for each species: “*Ca.* M. planktonicus”: KE-4b, “*Ca.* M. universalis”: GE-M14, ‘*Ca.* M. rimovensis: MMS-RI-41, OM43-b: LSUCC0717, OM43-c: LSUCC0568, OM43-d: LSUCC0268). Genomic islands [16] were identified by a reciprocal BLASTn between the genomes to analyze breaks in genome synteny and by using BLASTp (e-value thresholds of 1e-3) to compare each genome to all others within a species to pinpoint stretches of genes with recurring low alignment scores. Unique genes from each species were extracted (95% sequence identity threshold) using cd-hit-2d v4.6.6 [45]. A maximum likelihood tree of 87 conserved proteins of the tycheposon (Table S4) was constructed as outlined above (best fitting model JTTDCMut+F+R9). Integron finder v2.0 [46, 47] was used to identify integrons in the genomes. A maximum likelihood tree of aligned OstA (organic solvent tolerance protein) sequences of all genomes except for HTTC2181 was done with IQ-TREE [41] (best fitting model JTTDCMut+F+R9).

### Metagenomic sequencing and recruitment

DNA extraction and metagenomic sequencing (Illumina Novaseq 6000, PE150) of the 0.22-μm filters was done as previously described [48, 49]. Reads were quality controlled (Q > 33), adapter removed, and trimmed (trimq = 18 qtrim = rl) using BBMap v36.x [50]. Additionally, 61 seasonally resolved samples from the Řimov reservoir [49, 51], the TYMEFLIES dataset from Lake Mendota (94 samples collected over 5 years [52]), and 421 publicly available coastal and open ocean metagenomes [53-55] were used for fragment recruitment. Metagenomes were subsampled to 20 million reads, and rRNA genes in genomes were masked prior to recruitment. MMseqs [56] was used to map metagenomic reads to each individual genome and fGIs to obtain base coverage per Gb (-minid 0.95 -mincov 0.9 -minlen 50). Representative genomes of each species and specific metagenomes were selected to conduct fragment recruitments using parallel blat [57].

Temperature, pH, nitrate, and chlorophyll *a* values were used for Spearman correlations with fragment recruitment results of “*Ca.* Methylopumilus” genomes using R package Hmisc::rcorr [58]. Comparisons of metagenomic fragment recruitment results between different water layers (epi- vs. hypolimnion), lake types (oligo-, meso-, eutrophic lakes), and coverage of different fGIs were conducted using ANOVA (aov) and a TukeyHSD post hoc test [59].

### Growth experiments on the effect of decreasing methanol concentrations on “*Ca.* Methylopumilus”

Seven strains of “*Ca.* M. planktonicus” and 10 strains of “*Ca.* M. universalis” were grown in triplicates (25′000 cells ml^−1^ initial concentration) in media consisting of artificial lake water amended with vitamins and lanthanum chloride [21] with varying concentrations of methanol as sole carbon source (0, 0.0001, 0.001, 0.02, 0.05, 0.1, and 1 mM) at 16°C for 59 days until stationary phase. Subsamples were taken every 2 days until day 32, every 3–4 days thereafter, and a final measurement on day 59. Abundance assessment was done by flow cytometry as described above, and specific growth rates were calculated for each strain [60]. Comparisons of growth rates between subgroups were done using the Welch two sample t-test [59].

## Results

### Isolation success and genomic overview

We sequenced the genomes of 52 “*Ca.* Methylopumilus spp.” strains isolated from 14 different lakes in Central Europe and nine strains of the marine sister lineage OM43 isolated from coastal samples (Tables S1 and S3). These new genomes were analyzed together with 3 previously published OM43 [8, 29, 30] and 39 “*Ca.* Methylopumilus” [21] genomes. In total, 91 “*Ca.* Methylopumilus spp.” and 12 OM43 strains were used for genomic analyses. Most “*Ca.* Methylopumilus spp.” strains were isolated from the eutrophic Řimov reservoir (CZ, 21 strains plus 16 from [21]), followed by the mesotrophic Lake Zurich (CH, 3 strains plus 4 from [21]), mainly due to repeated isolation campaigns spanning several years [7, 21]. Half (53%) of the novel “*Ca.* Methylopumilus” strains originated from the epilimnion, similar to a previous isolation campaign at the Řimov reservoir [21] when 55% of the isolates originated from the epilimnion. Our isolation campaign, however, was still far from perfect in retrieving strains from all layers and all lakes. Rarely did the isolation result in strains from both layers of the same lake at the same time (except for Lake Greifensee). Moreover, lakes with high proportions of a certain “*Ca.* Methylopumilus” species would not always lead to its successful isolation. For example, we were unsuccessful in obtaining “*Ca.* M. planktonicus” cultures from Traunsee and Attersee although these lakes had the highest metagenomic fragment recruitment values for this species (see below).

**Figure 1:**
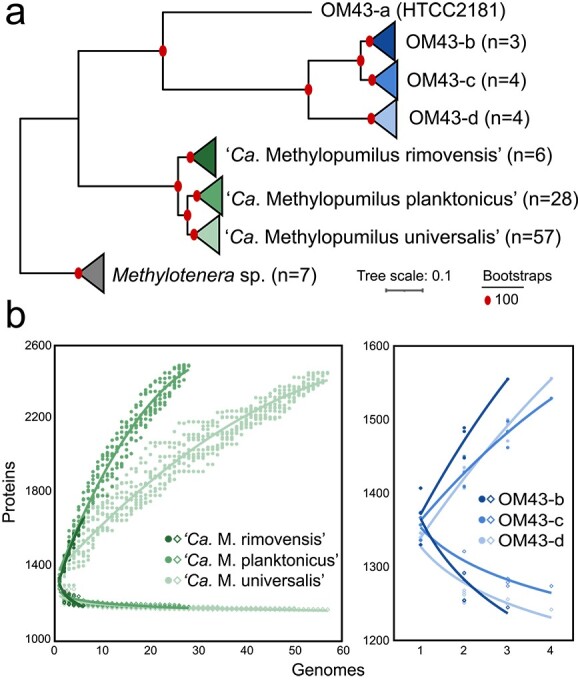
Phylogenomic and pangenome analyses of *Methylophilaceae*; (A) phylogenomic maximum likelihood tree of genomes used in this study; the tree was constructed with IQ-TREE with ultrafast bootstrapping using 851 common proteins; seven *Methylotenera* spp. genomes were used to root the tree; numbers in brackets indicate the number of genomes in each collapsed branch; a full version of this tree can be found as Fig. S1; (B) pangenome analysis of the three “*Ca.* Methylopumilus” and OM43 species. Symbols represent the core (diamonds) and pangenome (circles) on a species level; OM43-a was not included in this analysis due to the availability of only one genome.

All but one of the novel “*Ca.* Methylopumilus” strains clearly grouped with three previously proposed species [21] based on phylogenomic and ANI/AAI analyses using a 95% identity cut-off [37, 38] (Fig. 1A, Tables S5 and S6). The only exception was RI-55 that was loosely related to “*Ca.* Methylopumilus rimovensis” (93.1–94.8% ANI/AAI, Fig. S1, Tables S5 and S6). Overall, most strains were affiliated with “*Ca.* Methylopumilus universalis” (57 strains), which branched into two subgroups of strains isolated from Czech lakes and other countries, respectively, in the phylogenomic tree (Fig. S1), followed by “*Ca.* Methylopumilus planktonicus” (28 strains) and “*Ca.* Methylopumilus rimovensis” (5 strains plus RI-55, all exclusively isolated from eutrophic Řimov Reservoir). The nine new OM43 genomes represented mainly novel taxa that we named OM43-a-d (Fig. 1A and Fig. S1), with ANI/AAI values of 59–91% between different groups (Tables S5 and S6). Two new species were solely formed by our novel genomes (OM43-c and d), while one new strain (LSUCC0717) clustered with strains KB13 and M-MBSH7 (OM43-b) [29, 30], though below the species border (93–95% ANI/AAI). The first isolate of this lineage (HTCC2181) [8] formed a separate genus-level lineage (OM43-a) with AAI values <60% to all other strains, lower than the suggested threshold of 65–95% AAI [37].

Average genome sizes were 1.31 ± 0.03 Mbp for “*Ca.* M. planktonicus” and “*Ca.* M. rimovensis”, and 1.28 ± 0.02 Mbp for “*Ca. *M. universalis”, respectively, with GC contents of 37 ± 0.2% across all genomes. OM43 genomes averaged around 1.31 ± 0.02 Mbp with 35 ± 0.9% GC content (Table S3). Pangenome analysis of each “*Ca.* Methylopumilus” species revealed a stable core genome (1194, 1186, and 1171 common genes for “*Ca.* M. rimovensis”, “*Ca.* M. planktonicus”, and “*Ca*. M. universalis”, respectively) and an unsaturated pool of unique genes (1669, 2492, and 2452, respectively). OM43-b, c, and d contained 1245, 1274, and 1242 core genes and 1555, 1529, and 1556 auxiliary genes, respectively (Fig. 1B). An analysis was not possible for OM43-a as only a single genome was available.

### Abundances of different “*Ca.* Methylopumilus” and OM43 species in metagenomes

**Figure 2:**
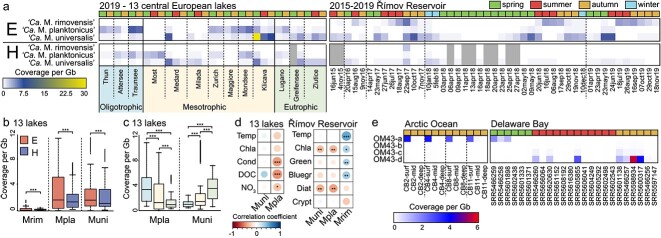
Occurrence of “*Ca.* Methylopumilus” and OM43 in metagenomic samples; (A) Metagenomic read recruitment (coverage per Gb at 95% identity) of “*Ca.* M. rimovensis,” “*Ca.* M. planktonicus,” and “*Ca. *M. universalis" in epi- and hypolimnetic samples of 13 central European lakes of different trophic states and seasonal samples of the Římov reservoir from 2015 to 2019. Different seasons are color-coded at the top. Mean values for each species are shown, and the full dataset can be found in Table S3; E: Epilimnion (5 m depth); H: Hypolimnion (variable depths, see Table S1); (B) coverage per Gb boxplots of individual species in epi- and hypolimnetic samples. Boxes indicate the 25th and 75th quantiles, medians are displayed by central lines, whiskers indicate the 5th and 95th quantiles, outliers are displayed by open circles; significant differences are displayed as asterisks (*P < .05; ** *P* < .01; ***P < .001); Mrim: “*Ca*. M. rimovensis”; Mpla: “*Ca.* M. planktonicus”; Muni: “*Ca. *M. universalis"; (C) coverage per Gb boxplots of individual species in oligo-, meso-, and eutrophic lakes. Trophic states are color-coded as in (A) and significant differences are displayed as asterisks as in (B); (D) Spearman correlations between metagenomic recruitment values and environmental factors across the 13 central European lakes and the Římov reservoir; significant differences are displayed as asterisks as in (B); Temp: water temperature (°C); Chla: chlorophyll *a* concentrations (μg l^−1^); Cond: conductivity (μS cm^−1^); DOC: dissolved organic carbon concentrations (mg l^−1^); NO_3_: nitrate concentrations (mg l^−1^); Green: chlorophyll *a* concentrations of green algae (μg l^−1^); Bluegreen: chlorophyll *a* concentrations of cyanobacteria (μg l^−1^); Diat: chlorophyll *a* concentrations of diatoms (μg l^−1^); Crypt: chlorophyll *a* concentrations of cryptophytes (μg l^−1^); (E) metagenomic read recruitment (coverage per Gb at 95% identity) of OM43-a, b, c, and d in samples of the Arctic Ocean and the Delaware Bay estuary.

The three freshwater “*Ca.* Methylopumilus” species showed species-specific patterns in fragment recruitment in metagenomic samples (Fig. 2A, Table S3). Generally, metagenomic recruitment was higher in epi- than in hypolimnetic samples for all three species (Fig. 2B). Exceptions were the three oligo-mesotrophic postmining lakes Most, Medard, and Milada, where most strains were almost below detection limit in the epilimnion (< 0.3 × coverage per Gb), but quantifiable in the hypolimnion (2–8 × coverage). “*Ca. *M. universalis" was most abundant in the mesotrophic Klíčava Reservoir (up to 31 × coverage) followed by the eutrophic Řimov Reservoir, oligo-mesotrophic Lake Medard, and eutrophic Greifensee and Žlutice Reservoir. In contrast, “*Ca.* M. planktonicus” recruited significantly more in less productive lakes (e.g. Traunsee, Thun, and Attersee; ANOVA, *P* < 2e-16; Fig. 2C), while “*Ca.* M. rimovensis” was mainly restricted to epilimnetic samples of the Řimov Reservoir (Fig. 2A).

Metagenomic fragment recruitment also showed that “*Ca.* M. planktonicus” was dominant in oligotrophic Lake Traunsee as the majority of reads recruited above 95% identity (Fig. S2). In contrast, “*Ca. *M. universalis" recruited reads mainly at around 90–94% identity in this lake, suggesting that related species were more prevalent. The opposite (i.e. recruitment at higher identity for “*Ca. *M. universalis" and lower identity for “*Ca.* M. planktonicus”) was observed for mesotrophic or eutrophic lakes (Fig. S2). These findings are supported by significant negative correlations of “*Ca.* M. planktonicus” abundances with parameters indicating a low trophic level (DOC, nitrate; Fig. 2D).

Clear seasonal trends were observed in multiannual samples of the Řimov Reservoir (81 samples from two depths spanning 5 years (Fig. 2A). Seasonal maxima of “*Ca. *M. universalis" in late spring and autumn (up to 12 × coverage) were followed by maxima of “*Ca.* M. rimovensis” in summer to early autumn (up to 9 × coverage). “*Ca.* M. planktonicus” was also present; however, it was never the dominant species (Fig. 2A, Fig. S3). Further analysis of environmental data confirmed these trends with significant positive correlations of “*Ca.* M. rimovensis” abundances with temperature and phytoplankton blooms (especially cyanobacteria and green algae), whereas negative trends with total chlorophyll *a* were observed for “*Ca.* M. universalis" (Fig. 2D). A similar seasonality of “*Ca.* M. universalis" was also identified in a 5-years metagenomic dataset from eutrophic Lake Mendota, USA [52], with annual maxima in late spring/early summer and autumn (Fig. S4).

Freshwater metagenomic recruitment against the four groups of marine OM43 yielded no coverage, which was also the case for samples from the open ocean [55] (Table S3). However, recruitment using Arctic Ocean [54] and estuarine [53] samples revealed an ecotype differentiation (Fig. 2E, Fig. S5). HTCC2181 (OM43-a) was present in surface samples of the Arctic Ocean (as shown before [54]), and OM43-d strains exhibited 5–6 × coverage in samples of Delaware Bay estuary during autumn and 1–2 × coverage during summer (Fig. 2D and Fig. S5). “*Ca.* Methylopumilus”, OM43-b, and c were not present in any of these samples, except for an occasional appearance of “*Ca*. M. universalis" and “*Ca*. M. rimovensis” in the Delaware Bay (up to 4 × coverage; Table S3).

### Flexible genomic islands in closely related *Methylophilaceae*

**Figure 3:**
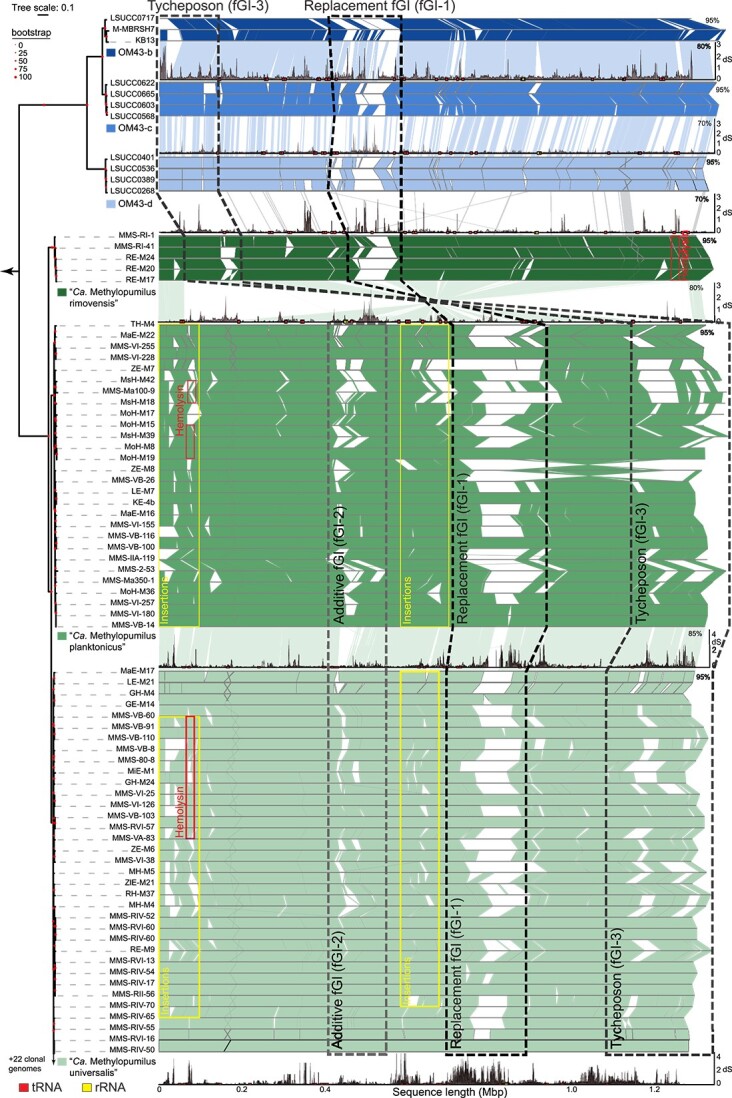
Flexible genomic islands (fGIs) in *Methylophilaceae*. Genomic alignment (BLASTn) of genomes following the phylogenomic tree from Fig. 1A; dS profiles of each species are shown below each alignment group using one genome as reference; the location of tRNA and rRNA genes are shown as squares; the approximate location of the three major fGIs are outlined as dashed boxes; locations of other insertion sites and the hemolysin cluster are displayed as boxes; *Methylotenera* spp., OM43-a (HTTC2181), “*Ca*. M. rimovensis” RI-55 were omitted from the analyses due to their high divergence, and 22 clonal genomes of “*Ca. *M. universalis" are not shown due to redundancy in their alignment; a full version including all genomes can be found as Fig. S6.

Fragment recruitment analyses also revealed gaps in mapped reads (Figs S2, S3, and S5) indicative of metagenomic islands [16]. Numerous gaps (95% identity threshold) were also present when aligning genomes of OM43 and “*Ca.* Methylopumilus” and the position of the largest gap (fGI-1) coincided with the metagenomic islands (Fig. 3, Fig. S6). In total, two large fGIs were identified in OM43 and “*Ca.* M. rimovensis” and three in “*Ca. *M. universalis" and “*Ca.* M. planktonicus”. The locations of fGIs were shared between the latter two species, except for ZE-M8 (“*Ca.* M. planktonicus”), where a part of the genome was inverted between the approximate Locations of fGI-1 and fGI-3 (Fig. 3). Major dS peaks were found for fGI-1 and fGI-3 with synonymous mutation rates ranging between 2 and 4 (Fig. 3, Fig. S7, Table S7). The synteny across the genomes in this study was highly conserved, with the exception of a large inversion in “*Ca.* M. rimovensis” and the OM43 group compared to “*Ca*. M universalis” and “*Ca*. M. planktonicus” including the origin of replication (*dnaA*). The synteny could be theoretically restored by reversing the inversion (Fig. S8), which would lead to an almost perfect alignment of fGI-1 and fGI-3 with “*Ca.* M universalis” and “*Ca.* M. planktonicus”. However, the rRNA operon in OM43 genomes—originally in synteny with the other rRNA operons in the freshwater species—would then align with fGI-2 of the latter.

**Figure 4:**
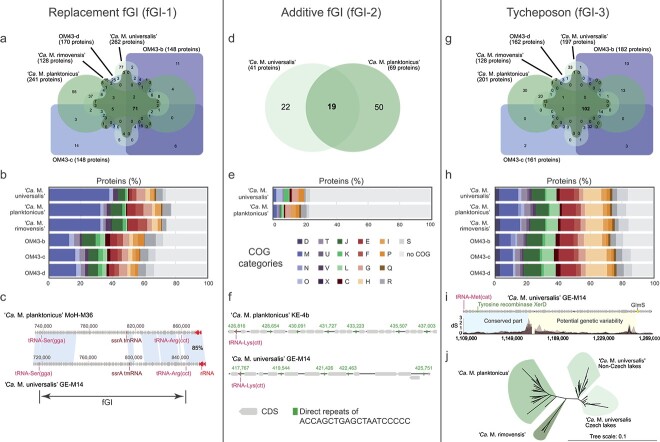
Common patterns of the different flexible genomic islands (fGIs); (A) Venn diagram of shared and unique proteins in the replacement fGI (fGI-1); (B) COG categories assigned to proteins in the replacement fGI of each species; (C) the replacement fGI is flanked by a tRNA-Ser(gga) gene on one end and by a tRNA-Arg(cct) on the other with a tmRNA gene in the middle of the island; this pattern is common to all “*Ca.* Methylopumilus”, OM43, and some *Methylotenera sp.* genomes; (D) Venn diagram of shared and unique proteins in the additive fGI (fGI-2); (E) COG categories assigned to proteins in the additive fGI of each species; (F) the additive fGI is identified by recurring repeats of the last 20 base pairs (ACCAGCTGAGCTAATCCCCC) of the tRNA-Lys(ctt) gene present in all “*Ca. *M. universalis" and “*Ca.* M. planktonicus” genomes; (G) Venn diagram of shared and unique proteins in the tycheposon (fGI-3); (H) COG category assigned to proteins in the tycheposon of each species; (I) the tycheposon starts with a tRNA-met(cat), contains tyrosine recombinase *xerD*, and ends at the first conserved gene (*glmS*—glucosamine 6-phosphate synthetase). It is common to all “*Ca.* Methylopumilus” and OM43 genomes and can be separated in a conserved part and a part with potential genetic variability; (J) a tree of 86 common proteins (see Table S2) of the tycheposon of “*Ca.* Methylopumilus” separates “*Ca.* M. universalis" in strains isolated from lakes in Czechia and other countries, respectively.

Closer investigations of each fGI revealed unique characteristics across the family (Table S9). The largest fGI (fGI-1) in all genomes represents a replacement fGI [16]. Sixteen % of the total protein pool in fGI-1 are shared among all species (Fig. 4A). Most genes of this fGI are either related to cell wall/membrane polysaccharide synthesis (COG category M: 32–38% for “*Ca.* Methylopumilus” and 13–17% for OM43) or with unknown function (no COG assigned and COG category S: 23–28% and 33–39% in “*Ca.* Methylopumilus” and OM43, respectively, compared to 17–19% across the whole genome for both groups; Fig. 4B). Additionally, one “*Ca.* M. planktonicus” strain (MMS-VI-228) contained genes encoding a sulfate transporter at the border of this island. A consequence of horizontal gene transfer via recombination typical for replacement fGIs is a high frequency of synonymous mutations at the borders, as visible in the dS profiles (Fig. 3, Table S8) and SNPs (Fig. S7). The replacement islands are flanked by tRNA genes (tRNA-Ser and tRNA-Arg) with a tmRNA gene in the middle, a pattern common to all “*Ca.* Methylopumilus”, OM43, and also two *Methylotenera* sp. genomes (Fig. 4C, Fig. S1).

The second largest fGI (fGI-2) was present only in “*Ca.* M. planktonicus” and “*Ca. *M. universalis”, located at around 0.4–0.5 Mbp (Fig. 3), where 20% of their contents are found in both species (Fig. 4D). SNP frequency was not elevated at the borders; however, direct repeats of the last 20 nucleotides of the tRNA-Lys (ACCAGCTGAGCTAATCCCCC) in varying frequencies precede these fGIs (Fig. 4F). These islands resemble additive fGIs with variable size (330 bp-42Kb; 1 to 63 CDS) and gene content [16] and possess a very high proportion of proteins with unknown function (79–80%, Fig. 4E; Table S9). Annotated functions were mainly related to COG categories P (inorganic ion transport and metabolism: 2–4%), G (carbohydrate transport and metabolism: 3–4%), and M (cell wall/membrane/envelope biogenesis: 2%). “*Ca*. M. universalis" additionally contained proteins related to COG categories O (posttranslational modification, protein turnover, and chaperones, 4%) and K (transcription, 3%). Several genes encoding for mechanisms involved in osmoregulation (*mscS*), metal transport, resistance and metabolism (*terC, bfr,* chromate ion transporter 2.A.51, *cirA*, TIGR00003, *czcD*), and oxidative stress (*msrA*) were identified in fGI-2. One strain each of “*Ca*. M. planktonicus” harbored a carbonic anhydrase and a nitroreductase (*nfnB*), respectively, and seven strains of *“Ca.* M. planktonicus” and two of “*Ca*. M. universalis" an additional photolyase (Table S9).

The third major fGIs (fGI-3), that were identified first in “*Ca.* M. planktonicus” and “*Ca. *M. universalis”, seem to be tycheposons, which are large cargo carrying transposons of 4–200 kbp length [61]. These fGIs are characterized by a tRNA encoding for methionine at their borders (one of the 7 tRNA genes described for *Prochlorococcus* tycheposons [61]) and by the presence of tyrosine recombinase *xerD* (Fig. 4I). Slight peaks in dS values but only few clear alignment gaps were observed for the tycheposons. Their content could be separated into a variable and nonvariable part, and they possessed the highest proportion of shared proteins across all fGIs (34%, Fig. 4G). Closer inspection of OM43 and “*Ca.* M. rimovensis” genomes revealed the presence of tycheposons with the same tRNA and recombinase genes (between 0.01–0.1 Mbp for OM43 and 0.07–0.2 Mbp for “*Ca.* M. rimovensis”, respectively) but in a reversed orientation. While tycheposons in *Prochlorococcus* seem to be involved in boosting nitrogen metabolism [61], no such clear trend was observed for *Methylophilaceae*. However, nitrogen, thiamine, and heme-related functions were present (*glnAK*, *gatABC*, *thiCDEGS, hemABEKL*), and other genes related to COG categories H (Coenzyme transport and metabolism: 11–14%), M (Cell wall/membrane/envelope biogenesis: 7–13%), J (translation, ribosomal structure, and biogenesis: 8–11%), E (amino acid transport and metabolism: 9–10%), and L (replication, recombination, and repair: 6–8%; Fig. 4H, Table S9). The number of proteins with unknown function in tycheposons was similar to the genome average (19–24%). Other notable genes encode for DNA recombination (*ruvABC*) and antimicrobial functionalities (*rhaT*, *mrcA*, COG5285, *salY, salX*). ANI values for the tycheposons of “*Ca. *M. universalis" and “*Ca.* M. planktonicus” (Table S10) were in some cases slightly lower than whole-genome ANI values although tycheposons partially harbored core genome genes in the nonvariable part. A phylogenomic tree of conserved proteins of the tycheposons (Table S2) revealed a separation of “*Ca.* M. universalis" into two subgroups of strains isolated from Czech lakes and other countries, respectively (Fig. 4J).

To further investigate the flexible genome’s potential influence, we conducted metagenomic recruitment for each fGI in “*Ca.* Methylopumilus” (Fig. S9, Table S11). OM43 was excluded due to low abundances in natural samples and fewer genomes per species. Tycheposons were highly abundant as they recruited significantly more reads than the other two fGIs (up to 29 × coverage, on average 86% of individual whole-genome recruitment values) and seem to be most conserved and common in natural populations. On the other extreme, the replacement fGI-1 recruited the least reads from metagenomes (up to 19 × coverage; 42% of whole-genome recruitments), indicating the highest variability and a high strain specificity. The additive fGI-2 occupied a place in-between the two former extremes (up to 24 × coverage, 55% of whole-genome recruitments; Fig. S9, Table S11).

Besides the three main fGIs, small insertions were detected all over the genomes (Fig. 3); however, none of these were *bona fide* integrons [47]. A high proportion of genes in these insertions could not be functionally annotated (48–68% for “*Ca.* Methylopumilus” and 67–72% for OM43; Table S9). The most common insertion was a cluster bordered by integrase *xerC* (occasionally also found in other fGIs; Table S9) with variable gene content. “*Ca*. M. universalis" MH-M5 included an insertion cluster with a complete type II secretion pathway operon (*gspCDEFLMGHIJKO)* associated with hypothetical proteins and a *sufI* multicopper oxidase potentially active during oxidative stress [62] and “*Ca*. M. planktonicus” ZE-M8 harbored one with an incomplete type IV pili (*pilABCDEMQTY*). Some strains encoded a thermostable hemolysin cluster (Fig. S1; Table S9). This cluster contained the same genes as described for *Vibrio cholerae* ICDC-VC702 (CP080463.1) but with protein identities ranging between 20 and 45%. Hemolysin in *Vibrio* spp. was previously described as a virulence agent lysing erythrocytes [63]. Further, most “*Ca.* Methylopumilus” genomes harbored a degraded vibriophage-like prophage (Bacteriophage KVP40, present in 54 “*Ca. *M. universalis”, 27 “*Ca.* M. planktonicus,” and 4 “*Ca.* M. rimovensis” genomes, respectively).

### Growth of “*Ca*. Methylopumilus” strains at different methanol concentrations reveals further ecotype differentiation

**Figure 5:**
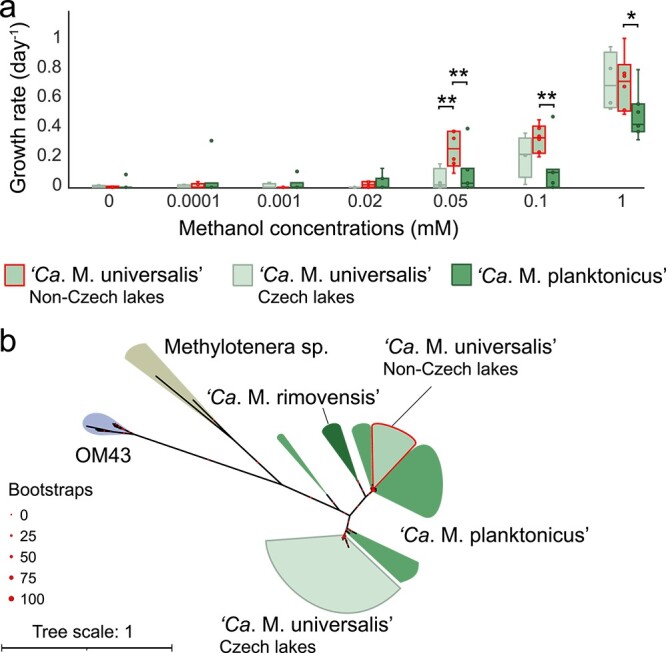
Growth of “*Ca.* Methylopumilus” influenced by different methanol concentrations; (A) growth rates (day^−1^) of seven strains of “*Ca.* M. planktonicus” and 10 strains of “*Ca.* M. universalis” (4 strains isolated from lakes in Czechia and 6 strains from other countries) under increasing methanol concentrations (0, 0.0001, 0.001, 0.02, 0.05, 0.1 and 1 mM); significant differences are indicated by asterisks (*P < .05; **P < .01); individual growth curves are shown in Fig. S10; (B) maximum likelihood tree of OstA (organic solvent tolerance protein) of *Methylophilaceae*; the tree was constructed with IQ-TREE with ultrafast bootstrapping; OM43-a HTCC2181 was excluded due to high sequence divergence.

To further identify potential ecophysiological differences within *“Ca.* Methylopumilus”, growth of 7 “*Ca.* M. planktonicus” and 10 “*Ca. *M. universalis" strains was monitored in response to different methanol concentrations (0.1 μM to 1 mM, Fig. 5A, Fig. S10). No growth occurred at concentrations <20 μM methanol, and no significant differences were detected between “*Ca.* M. planktonicus” and “*Ca.* M. universalis" strains (Fig. 5A). However, a closer inspection of the two subgroups within “*Ca.* M. universalis" identified in phylogenomic trees of the core-genome and the tycheposon (Fig. 4J, Fig. S1) revealed differences in growth rates at 50 μM methanol. The subgroup containing only strains isolated from Czechia showed no growth (except for slight growth of MH-M5), while strains from other locations did grow (Welch two sample t-test, *t* = −3.332, *P* = .021 between Czech and non-Czech strains). Moreover, growth rates of the non-Czech strains were significantly higher than “*Ca.* M. planktonicus” at concentrations >50 μM methanol (Fig. 5A, Fig. S10). Further genomic inspections of the two subgroups of “*Ca.* M. universalis" revealed a variation in the *ostA* gene (organic solvent tolerance protein). A phylogenetic tree resulted in a clear separation of strains growing at low methanol concentrations (Fig. 5B). This essential membrane protein is crucial for the permeability of carbon compounds much larger than methanol (diphenyl-ether) in *Escherichia coli* [64-66] and might be responsible for the observed growth differences at very low substrate concentrations.

## Discussion

### Niche differentiation of *Methylophilaceae* on species and ecotype levels

Seasonal epilimnetic maxima of “*Ca*. Methylopumilus” were previously reported for late spring and autumn-winter [7, 18, 67]. Negative correlations of natural populations with water temperature were confirmed in growth experiments of one strain (“*Ca*. M. planktonicus” MMS-2-53 [7]). Other studies however, found distinct associations of “*Ca.* Methylopumilus” with summer cyanobacterial blooms [68, 69]. These two different dynamics might be driven by different “*Ca.* Methylopumilus” species present in different lake types and seasons. Maxima in spring and autumn-winter are likely indicative of “*Ca. *M. universalis" or “*Ca*. M. planktonicus” proliferation, while “*Ca.* M. rimovensis” seems to be associated with algal and cyanobacterial blooms in summer (Fig. 2, Figs S2–S4). Moreover, “*Ca.* M. planktonicus” is more abundant in oligotrophic lakes, while “*Ca.* M. universalis" mainly inhabits meso- to eutrophic lakes (Fig. 2). We identified a similar ecotype separation in the marine OM43 clade (Fig. 2D and Fig. S5), with strain HTTC2181 being more common in cold, coastal arctic waters [54] and OM43-d strains in temperate coastal samples. OM43-b were previously reported in warm low-chlorophyll *a* provinces such as the Arabian Sea [30]; however, they were not detected in the TARA-oceans data [55]. The preferred habitat of OM43-c is currently unknown. Overall, the clear species-specific habitat preferences regarding trophic levels (oligo- vs. eutrophic lakes), water temperature, primary producers (co-occurrence vs. avoidance), and seasonal changes in the same system (spring and autumn maxima vs. summer maxima) point to specific ecophysiological adaptations and highlight the importance to differentiate between closely related species in environmental surveys.

Another level of ecotype differentiation and potential biogeography within “*Ca*. M. universalis" was apparent from experiments in methanol utilization and further genomic analyses (Fig. 5). We hypothesize that different variants of the organic solvent tolerance protein OstA are responsible for differences in membrane permeability of methanol at low concentrations, similar to reduced/enhanced permeability of organic solvents in *E. coli* [64-66]. This membrane protein might be potentially crucial for ecophysiological adaptations in OM43 as well, as *ostA* was highly expressed in OM43 when grown with 50 μM methanol compared to more complex carbon sources [70].

### Flexible genomic islands supporting a habitat transition

Ecophysiological adaptations are often found in the flexible genome, explaining ecotype differentiation [16]. Numerous studies have investigated microdiversity in microorganisms, e.g. in SAR11 [5, 71, 72], *Alteromonas macleodii* [73], *Vibrio vulnificus* [74], and other taxa [12, 75-79]. However, genomic microdiversity analyses spanning multiple genera from different habitats are rare. The *Methylophilaceae* family offers an interesting outlook due to the freshwater-marine habitat transition described for “*Ca.* Methylopumilus” and OM43 [21]. Much of the genomic characteristics of freshwater “*Ca.* Methylopumilus” are shared with the marine sister clade OM43, such as similar genome and core genome size (Fig. 1), GC content, and metabolic pathways [21]. There is clear evidence of conserved patterns in flexible genome organization from “*Ca. *Methylopumilus” to the OM43 clade and sometimes even to *Methylotenera* spp. (Fig. 4, Fig. S1). Moreover, two fGIs (replacement fGI-1 and tycheposon fGI-3) possess similar patterns (same tRNA genes flanking or preceding fGIs and similar gene content) in both taxa (Figs 3 and 4) and even the locations coincide except for a large inversion in OM43 and “*Ca.* M. rimovensis” compared to the other two taxa (Fig. S8). The inversion might have been caused by the integration of the tycheposon and hint at potentially fixed chromosomal loci for genomic variability. These genomic similarities further support a freshwater to marine habitat transition [21] where the last common ancestor of “*Ca*. Methylopumilus” and OM43 seems to have acquired fGI-3 and different, constantly changing, variants of fGI-1, while fGI-2 was gained during a later differentiation phase of “*Ca*. M. universalis" and “*Ca*. M. planktonicus”.

The three main fGIs differed in their relative abundance across our metagenomes (Fig. S9, Table S11) with the tycheposon (fGI-3) being most common in natural populations, while fGI-1 appears to be more strain specific. A duplication of the nitrate assimilation function in the tycheposon of *Prochlorococcus* required long selection pressure in culture conditions while the rest of the genome remained unchanged [61]. Likewise, persistent environmental selection pressure might have led to the stable integration of fGI-3 in the last common ancestor of “*Ca*. Methylopumilus” and OM43. On the other hand, naturally occurring constantly high selection pressure by phage predation leads to rapid coevolution of both phages and hosts that require constant turnover of receptor sites [80-83]. This might explain the observed high variability in fGI-1, which can be regarded as the de facto agent for phage evasion by glycocalyx modification [15].

### Selective advantages aided by the flexible gene pool

The presence of replacement and additive fGIs in *Methylophilaceae* was expected given that these are commonly found across prokaryotic life and governed by essential processes of recombination and activity of mobile genetic elements [15, 84-90]. What was unexpected, however, was the discovery of tycheposons in genomes of “*Ca*. Methylopumilus” and OM43. This type of flexible genomic element has only been discovered recently in marine *Prochlorococcus* and other taxa [61]. In *Prochlorococcus*, cargo-carrying tycheposons mainly act in nutrient uptake and nitrate assimilation [61], and similarly, genes for nitrogen metabolism were found in “*Ca.* Methylopumilus” (Table S9). Further, genes for heme and thiamine biosynthetic pathways and cytochrome B561 were located in the tycheposons of *“Ca*. Methylopumilus” and OM43. This might give them competitive advantages in nutrient-deprived habitats such as the pelagial of lakes and coastal oceans. While the role of the replacement fGI (fGI-1) seems to be clearly related to the avoidance of phage predation [15], the dominance of hypothetical proteins in fGI-2 makes function predictions challenging (Fig. 4, Table S9). However, we identified several common proteins such as photolyases, dehydrogenases, and membrane transporters as well as species- and strain-specific ones (e.g. nitroreductase, carbonic anhydrase, azurin, different cytochromes, resistance genes, NAD/NADP transhydrogenases). Thus, the repertoire of annotated accessory genes in fGI-2 mirrors published functions such as resistance to heavy metals, transporters, UV repair, and other metabolic functions that confer selective advantages to individual strains [12, 16, 73, 77, 85].

## Conclusion

Our study revealed high levels of genomic microdiversity within the family *Methylophilaceae* that was organized very similarly across genera from different habitats. Each species is adapted to a distinct niche depending on the season, trophic status, or location. Despite these differences, the genome synteny and potential role of each fGI was relatively conserved across both freshwater “*Ca.* Methylopumilus” and marine OM43. The three main fGIs differed in their metagenomic recruitment suggesting different levels of variability and hence different ecological roles ranging from strain-specific to population-level adaptations. Moreover, variants in one gene (*ostA*) seem to trigger ecotype differentiation in the growth response at low substrate concentrations and hint at a potential biogeographic segregation of one species. Whether or not other genetic components (e.g., tycheposons) play a role in niche separation and biogeographic distribution of *Methylophilaceae* remains to be tested in further experiments on strains isolated from lakes spanning a larger geographic region.

## Supplementary Material

Layoun_Supplementary_material_wrad036

Layoun_Suppl_tables_wrad036

## Data Availability

All freshwater data used in this study have been submitted to ENA under project accession numbers PRJEB35640, PRJNA429141 (metagenomes of the 13 lakes and the Řimov Reservoir, respectively), and PRJEB53432 (genomes). Genomes of OM43 isolates have been submitted to NCBI under project accession number PRJNA857682.
