# Supplementary material for: Flexible genomic island conservation across freshwater and marine Methylophilaceae
Source: ISME J. 2024 Jan 10;18(1):wrad036. doi: 10.1093/ismejo/wrad036 (PMC10872708; doi:10.1093/ismejo/wrad036)
Supplement: Layoun_Supplementary_material_wrad036 [file layoun_supplementary_material_wrad036.pdf]

Supplementary material to

Flexible genomic island conservation across freshwater and marine *Methylophilaceae*

Paul Layoun[1,2], Mario López-Pérez[3], Jose M. Haro-Moreno[3], Markus Haber[1], J. Cameron Thrash[4], Michael W. Henson[5], Vinicius Silva Kavagutti[1,2], Rohit Ghai[1], Michaela M. Salcher[1*]

Supplementary Figures:

Figure S1: Full phylogenomic tree from Fig. 1a and Fig. 3.
Figure S2: Metagenomic fragment recruitment with samples from lakes of different trophic states.
Figure S3: Metagenomic fragment recruitment with samples from different seasons.
Figure S4: Metagenomic read recruitment of '*Ca.* Methylopumilus' in seasonal samples of Lake Mendota.
Figure S5: Metagenomic fragment recruitment with samples from the Arctic Ocean and Delaware Bay.
Figure S6: Genomic alignment (BLASTn) of all genomes including 22 clonal genomes of '*Ca.* M. universalis' as displayed in Fig. 3.
Figure S7: Single nucleotide polymorphism (SNP) density plot of each '*Ca.* Methylopumilus' and OM43-b, c and d genome.
Figure S8: Genomic alignment of OM43 and 'Ca. Methylopumilus' after artificial sequence reversal of OM43 and 'Ca. Methylopumilus rimovensis'.
Figure S9: Metagenomic fragment recruitment boxplot (coverage per gb) of each fGI in '*Ca.* Methylopumilus'.
Figure S10: Individual growth curves of 10 '*Ca.* Methylopumilus universalis' and 7 '*Ca.* Methylopumilus planktonicus' isolates in triplicates under increasing methanol concentrations.

Supplementary tables can be found as separate xlsx file (Layoun_Suppl_Tables.xlsx)

Supplementary Tables:

Table S1: Sampling sites, details for isolates and metagenomes, and physicochemical data
Table S2: Primers designed for closing genomes
Table S3: Genome statistics and metagenomic fragment recruitment data
Table S4: Marker proteins (TIGR annotation) used for phylogenomic trees in Fig. 1, Fig. 3, and Fig. S1
Table S5: Average nucleotide identities (ANI) between all genomes
Table S6: Average amino acid identities (AAI) between all genomes
Table S7: Single nucleotide polymorphism (SNP) analysis
Table S8: Profiles of synonymous mutations rates (dS)
Table S9: Gene content of the three main flexible genomic islands (fGIs) and the insertions
Table S10: Average nucleotide identities (ANI) of tycheposons
Table S11: Metagenomic fragment recruitment of the three main flexible genomic islands (fGIs) in 'Ca. Methylopumilus'.

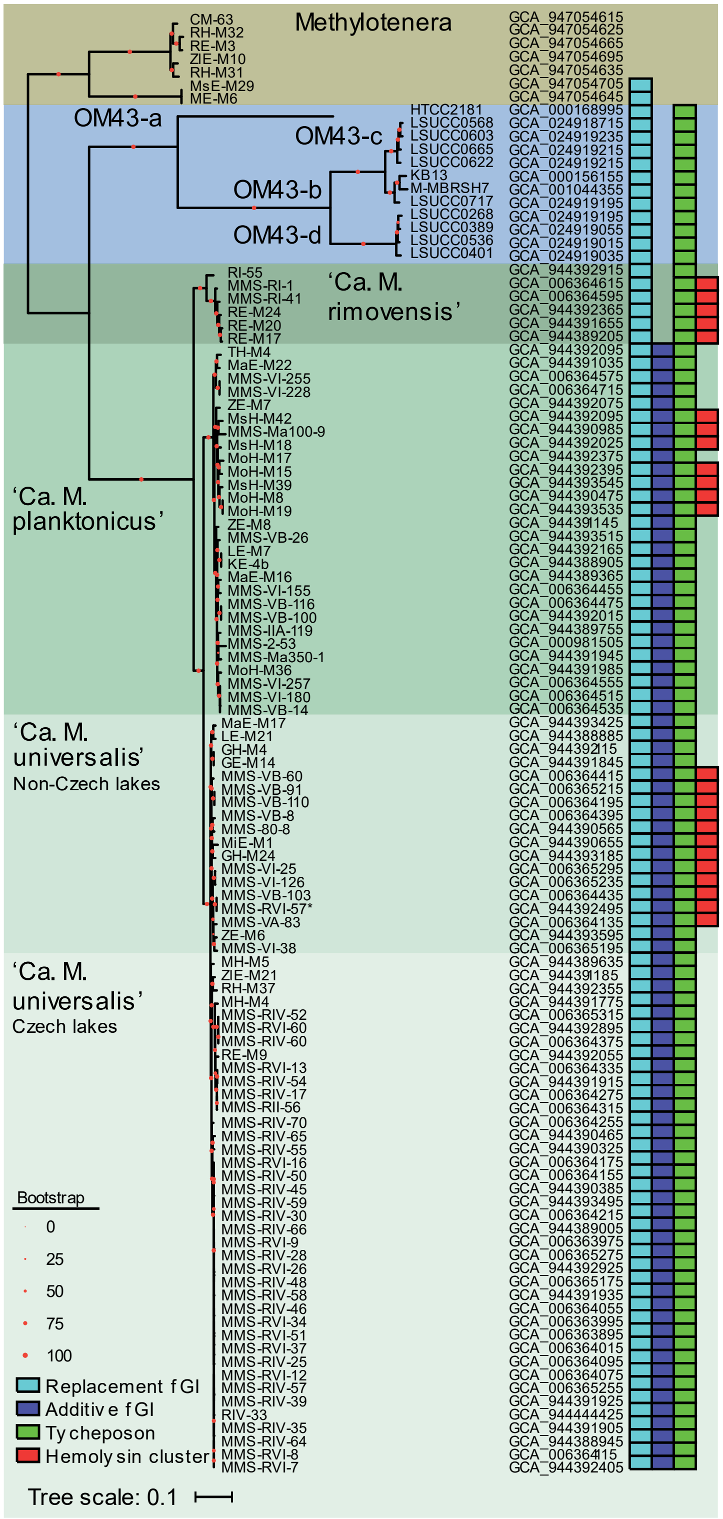

Figure S1: Full phylogenomic tree from Fig. 1a and Fig. 3. Phylogenomic maximum likelihood tree of genomes used in this study. The tree was constructed with IQ-TREE with ultrafast bootstrapping using 851 marker proteins. Seven *Methylotenera* spp. genomes were used to root the tree. Presence of different fGIs and the hemolysin cluster are indicated on the right next to genome accession numbers.

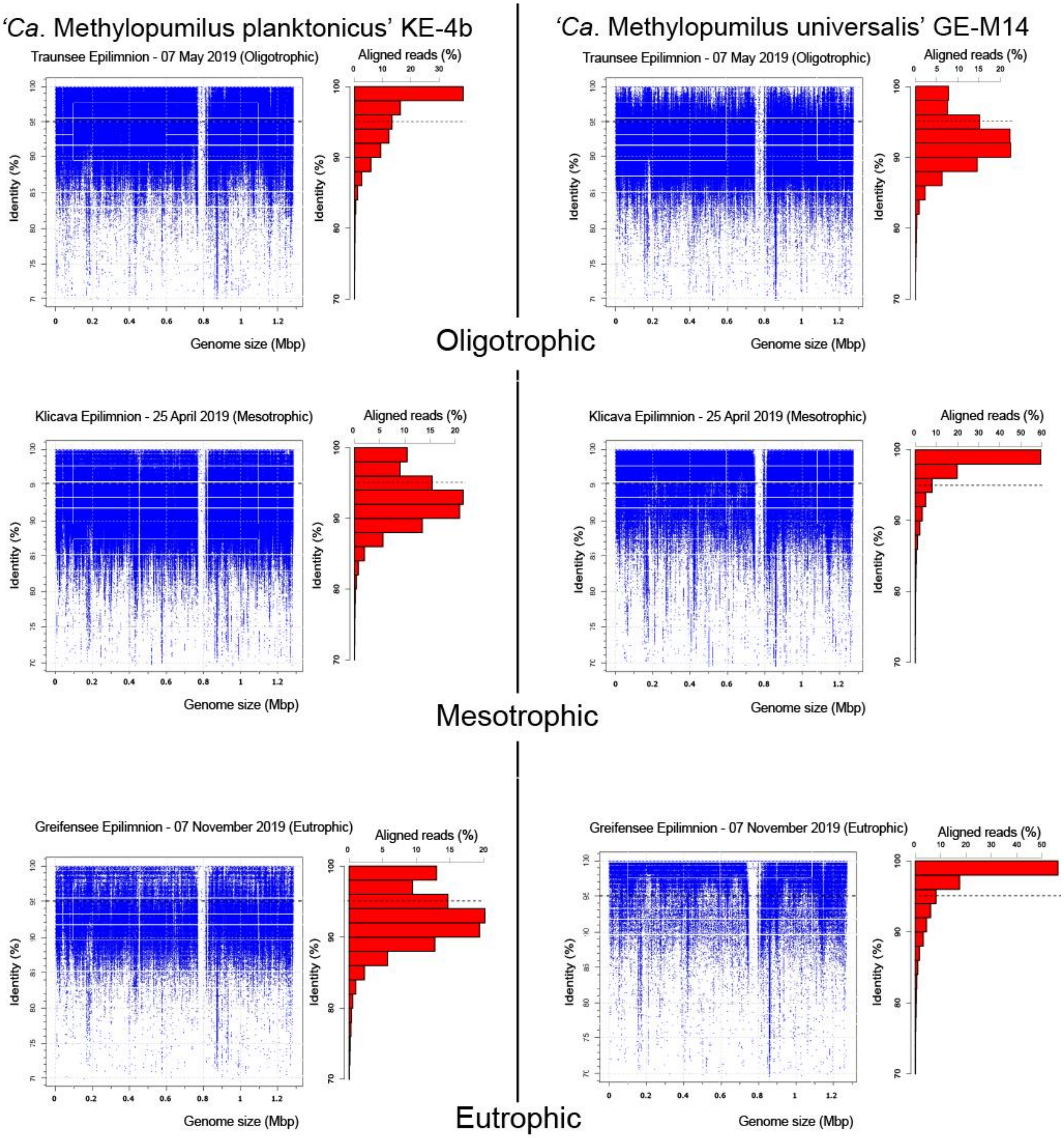

Figure S2: Metagenomic fragment recruitment with samples from lakes of different trophic states. Mapping of metagenomic reads from oligotrophic (Traunsee), mesotrophic (Klicava) and eutrophic (Greifensee) lakes to representative genomes of '*Ca*. Methylopumilus planktonicus' (KE-4b) and '*Ca*. Methylopumilus universalis' (GE-M14) using parallel blat. The dashed lines indicate 95% sequence identity. Bar charts on the right display proportions of mapped reads in 2% sequence identity increments.

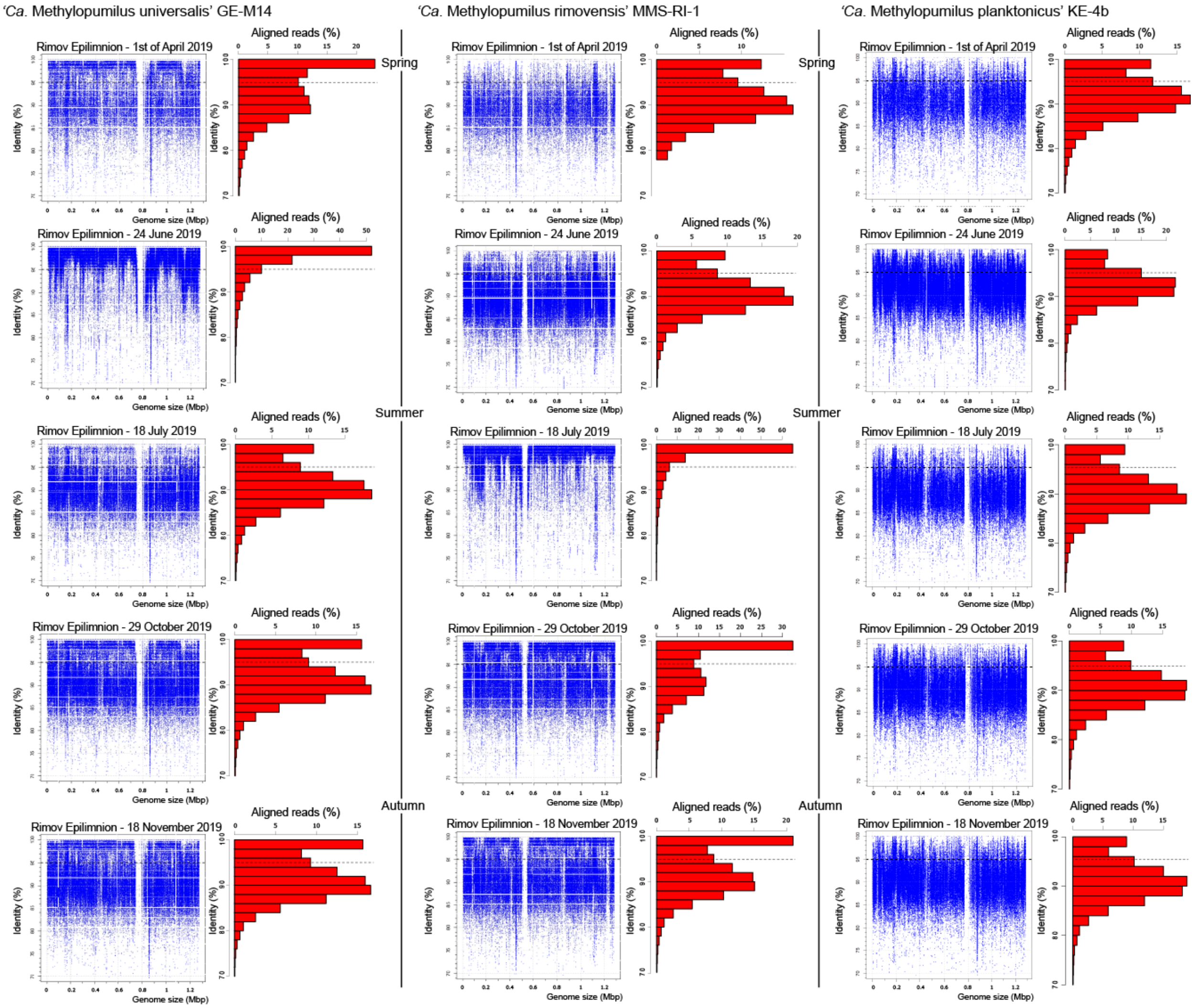

Figure S3: Metagenomic fragment recruitment with samples from different seasons. Mapping of metagenomic reads from Římov reservoir taken at different seasons in 2019 to representative genomes of '*Ca*. Methylopumilus planktonicus' (KE-4b), '*Ca*. Methylopumilus universalis' (GE-M14), and '*Ca.* Methylopumilus rimovensis' (MMS-RI-1) using parallel blat. The dashed lines indicate 95% sequence identity. Bar charts on the right display proportions of mapped reads in 2% sequence identity increments.

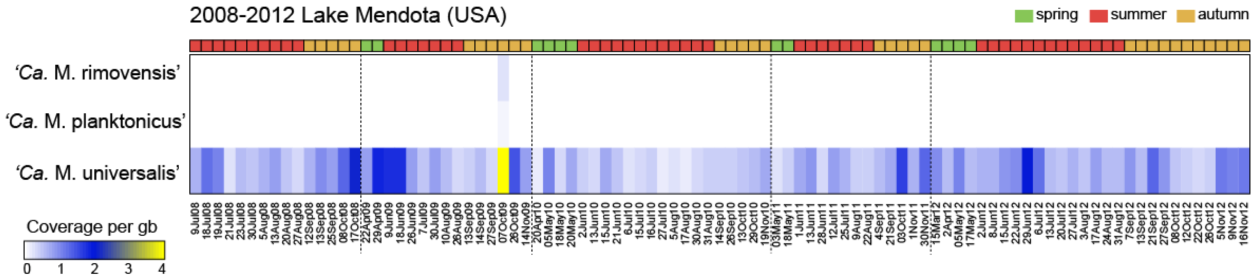

Figure S4: Metagenomic read recruitment of '*Ca.* Methylopumilus' in seasonal samples of Lake Mendota. Metagenomic read recruitment (coverage per gb at 95% identity) of '*Ca.* M. rimovensis', '*Ca.* M. planktonicus' and '*Ca.* M. universalis' in epilimnetic seasonal samples of Lake Mendota (USA) from 2008-2012. Different seasons are color-coded at the top. Mean values for each species are shown, the full dataset can be found in Table S3.

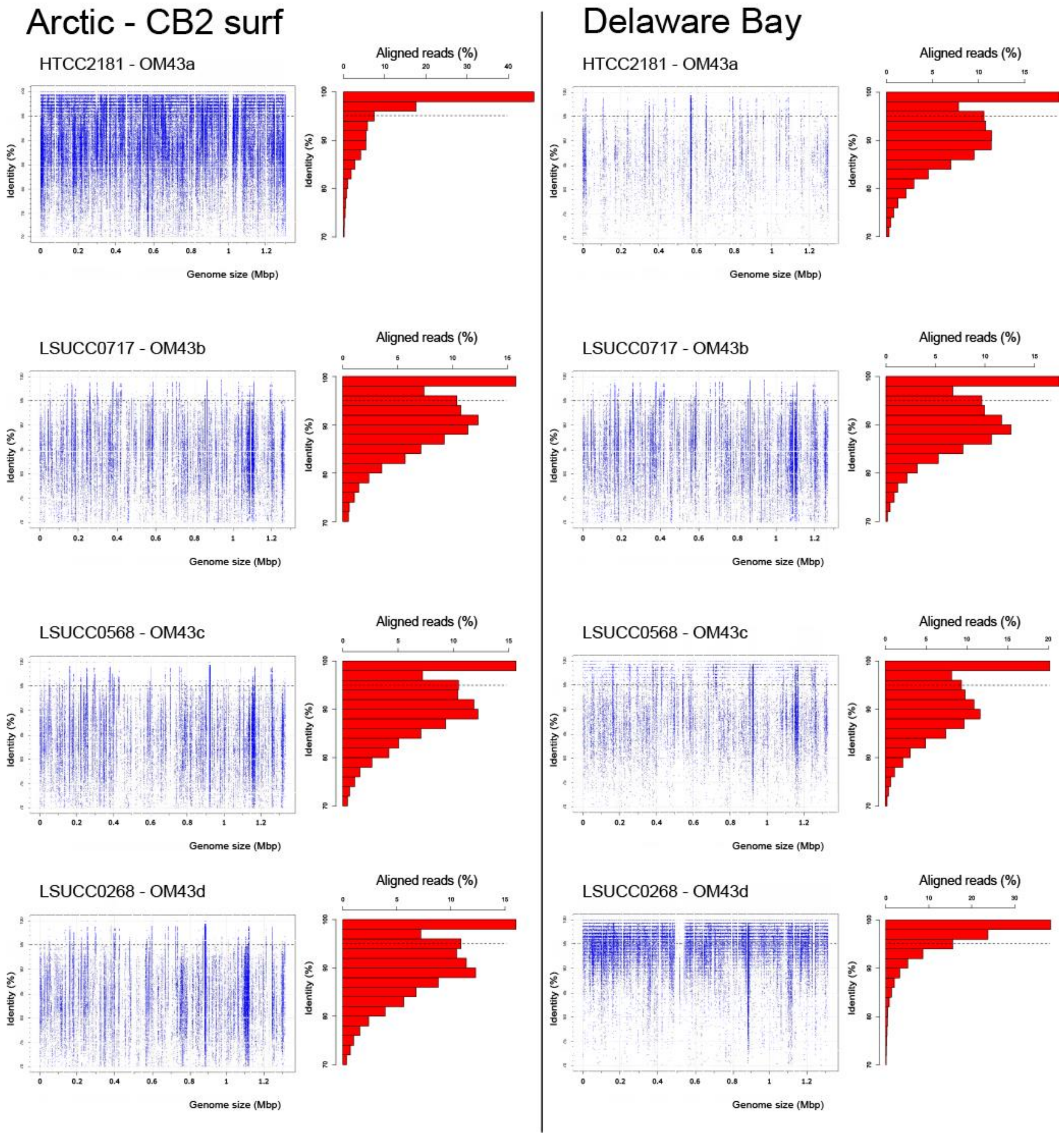

Figure S5: Metagenomic fragment recruitment with samples from the Arctic Ocean and Delaware Bay. Mapping of metagenomic reads from the Arctic Ocean and Delaware Bay estuary to representative genomes of OM43-a (HTCC2181), b (LSUCC0717), c (LSUCC0568) and d (LSUCC0268) using parallel blat. The dashed lines indicate 95% sequence identity. Bar charts on the right display proportions of mapped reads in 2% sequence identity increments.

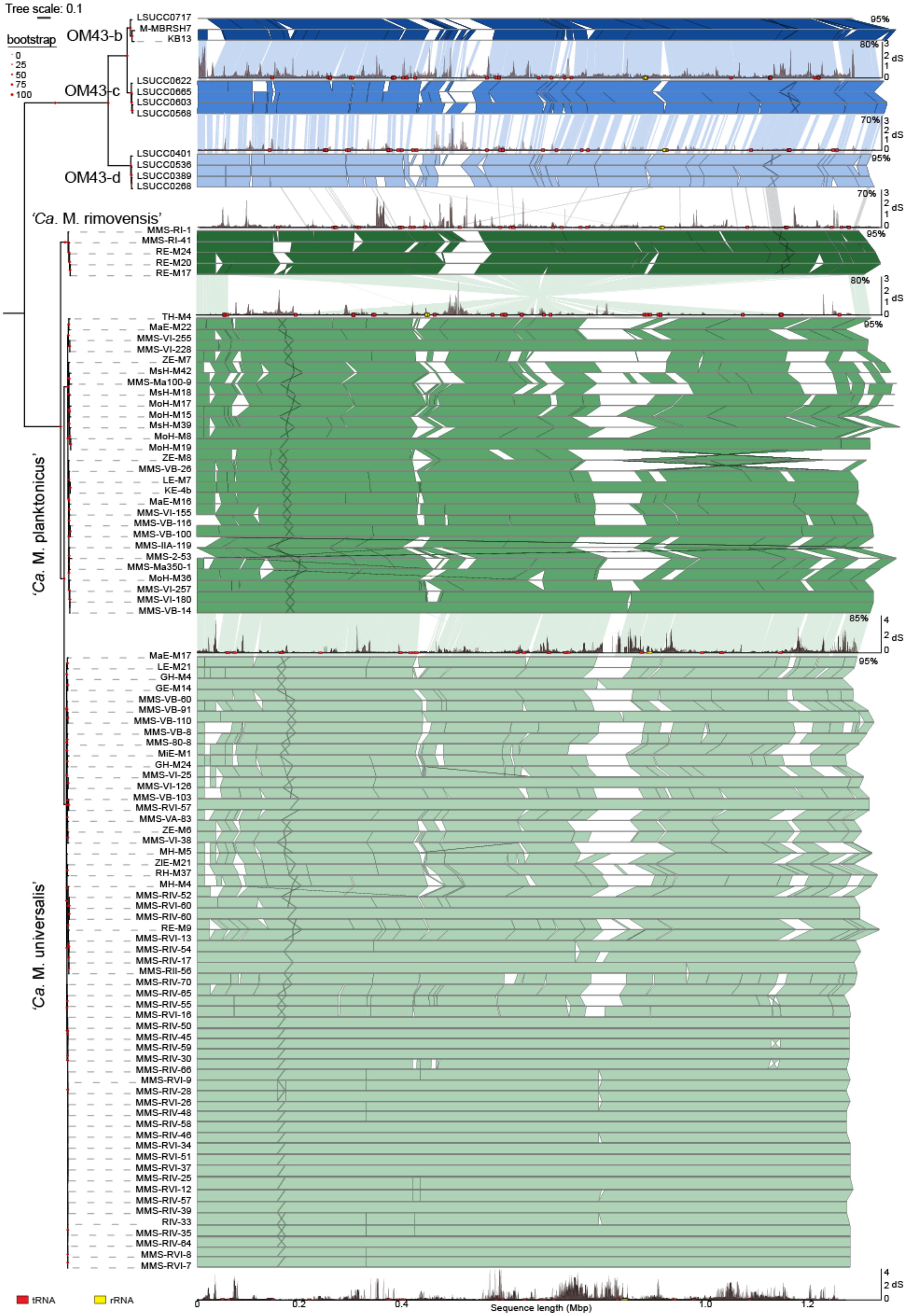

Tree scale: 0.1
bootstrap
0
25
50
75
100
OM43-b
OM43-c
OM43-d
'Ca. M. rimovensis'
'Ca. M. planktonicus'
'Ca. M. universalis'
LSUCC0717
M-MBRSH7
KB13
LSUCC0622
LSUCC0665
LSUCC0603
LSUCC0568
LSUCC0401
LSUCC0536
LSUCC0389
LSUCC0268
MMS-RI-1
MMS-RI-41
RE-M24
RE-M20
RE-M17
TH-M4
MaE-M22
MMS-VI-255
MMS-VI-228
ZE-M7
MsH-M42
MMS-Ma100-9
MsH-M18
MoH-M17
MoH-M15
MsH-M39
MoH-M8
MoH-M19
ZE-M8
MMS-VB-26
LE-M7
KE-4b
MaE-M16
MMS-VI-155
MMS-VB-116
MMS-VB-100
MMS-IIA-119
MMS-2-53
MMS-Ma350-1
MoH-M36
MMS-VI-257
MMS-VI-180
MMS-VB-14
MaE-M17
LE-M21
GH-M4
GE-M14
MMS-VB-60
MMS-VB-91
MMS-VB-110
MMS-VB-8
MMS-80-8
MiE-M1
GH-M24
MMS-VI-25
MMS-VI-126
MMS-VB-103
MMS-RVI-57
MMS-VA-83
ZE-M6
MMS-VI-38
MH-M5
ZIE-M21
RH-M37
MH-M4
MMS-RIV-52
MMS-RVI-60
MMS-RIV-60
RE-M9
MMS-RVI-13
MMS-RIV-54
MMS-RIV-17
MMS-RII-56
MMS-RIV-70
MMS-RIV-65
MMS-RIV-55
MMS-RVI-16
MMS-RIV-50
MMS-RIV-45
MMS-RIV-59
MMS-RIV-30
MMS-RIV-66
MMS-RVI-9
MMS-RIV-28
MMS-RVI-26
MMS-RIV-48
MMS-RIV-58
MMS-RIV-46
MMS-RVI-34
MMS-RVI-51
MMS-RVI-37
MMS-RIV-25
MMS-RVI-12
MMS-RIV-57
MMS-RIV-39
RIV-33
MMS-RIV-35
MMS-RIV-64
MMS-RVI-8
MMS-RVI-7
95%
80%
70%
95%
70%
95%
80%
95%
85%
95%
dS
0
1
2
3
4
tRNA
rRNA
0
0.2
0.4
1.0
1.2
Sequence length (Mbp)

Figure S6: Genomic alignment (BLASTn) of all genomes including 22 clonal genomes of '*Ca.* M. universalis' as displayed in Fig. 3. The location of tRNA and rRNA genes are shown as red and yellow squares, respectively, identity scores are given on the right side.

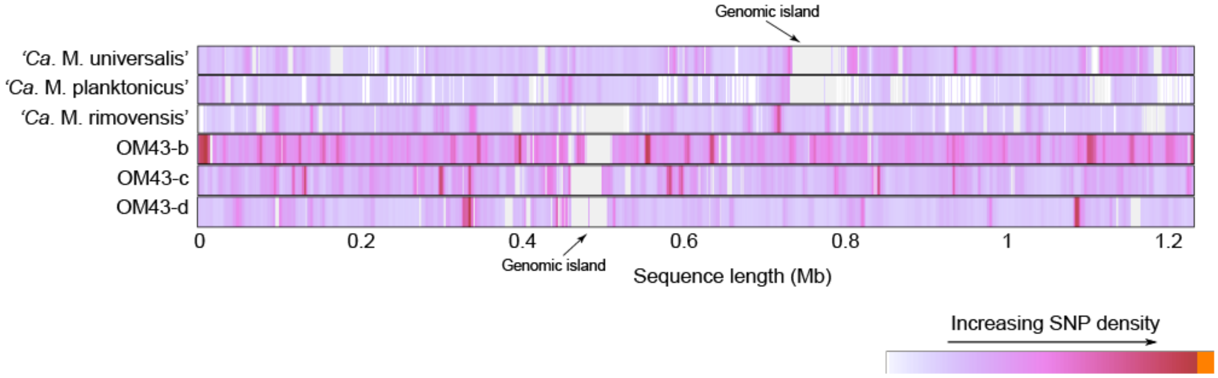

Figure S7: Single nucleotide polymorphism (SNP) density plot of each '*Ca.* Methylopumilus' and OM43-b, c and d genome.

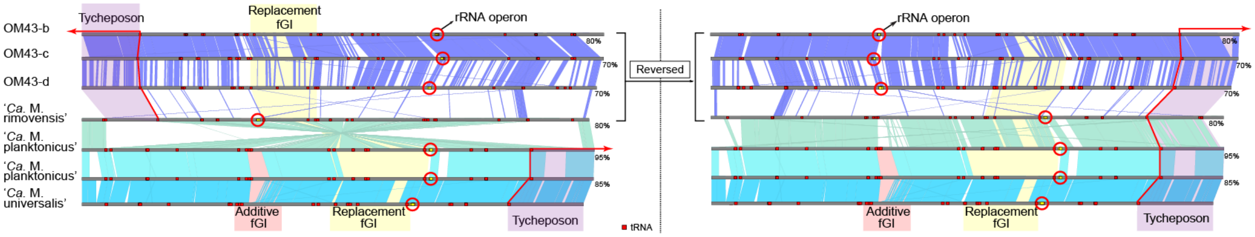

Figure S8: Genomic alignment of OM43 and 'Ca. Methylopumilus' after artificial sequence reversal of OM43 and 'Ca. Methylopumilus rimovensis'. Genomes were artificially reversed to demonstrate a preservation of synteny and the location of major fGIs, tRNA and rRNA genes.

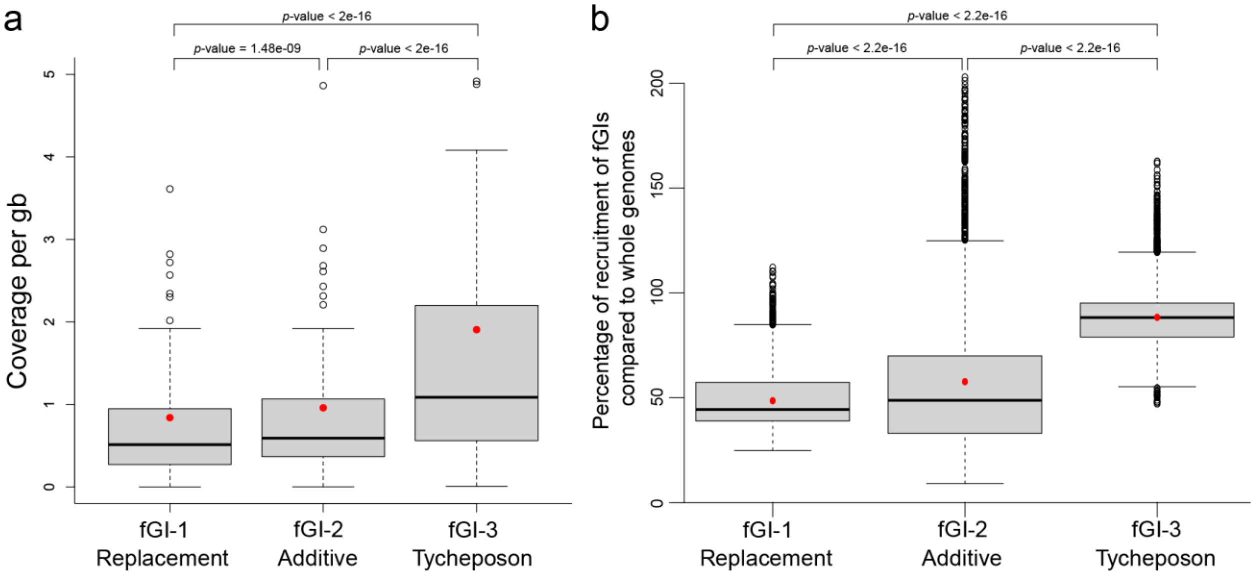

Figure S9: Metagenomic fragment recruitment boxplot of each fGI in '*Ca.* Methylopumilus' displayed as coverage per Gb (a) and proportion of whole genome recruitment values (b). Significant differences between fGIs are indicated by *p*-values, the full dataset can be found in Table S11. Boxes indicate the 25th and 75th quantile, medians are displayed by bold lines, means by red circles, whiskers indicate the 5th and 95th quantile, outliers are displayed by open circles.

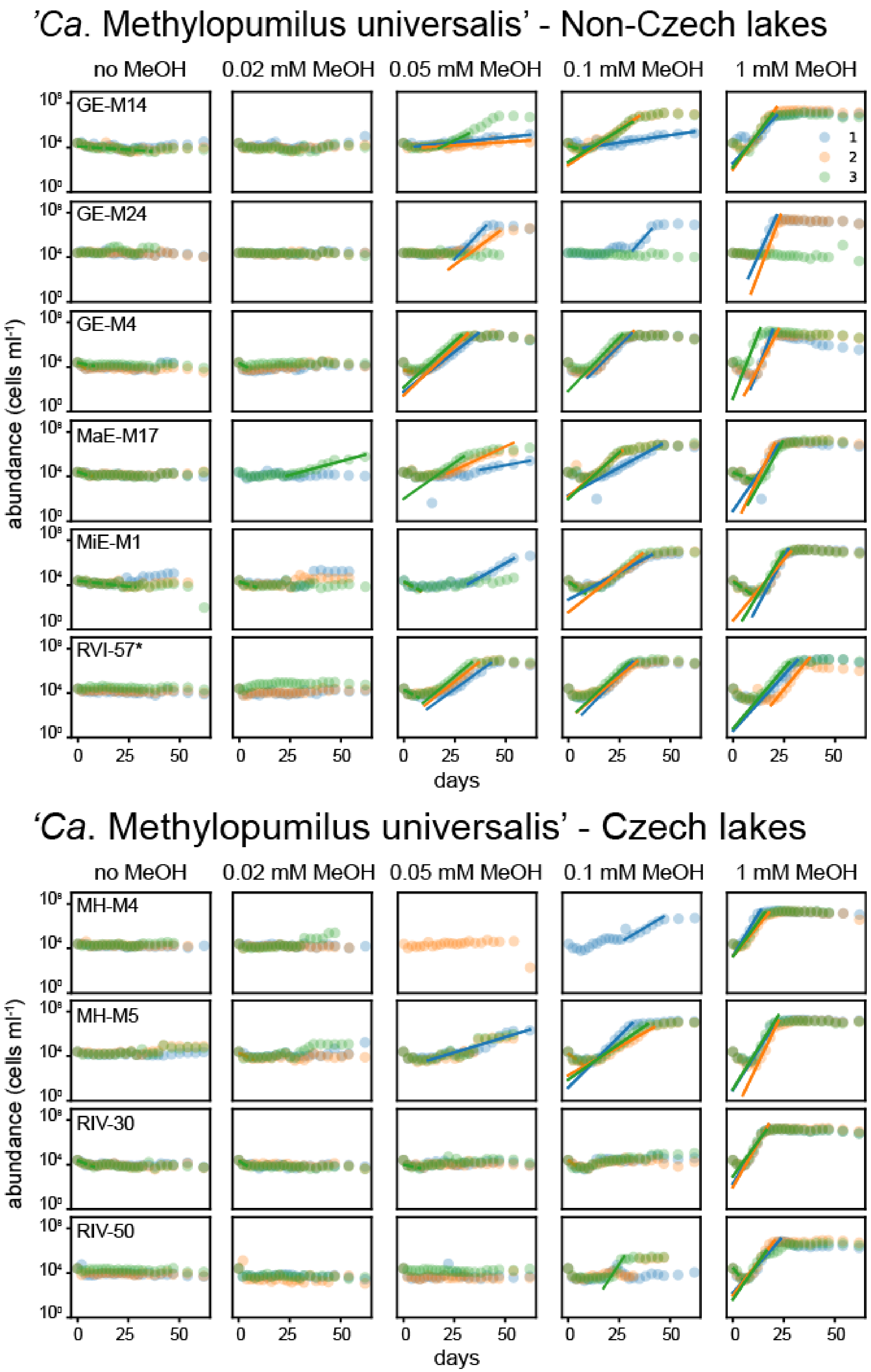

'Ca. Methylopumilus universalis' - Non-Czech lakes
no MeOH
0.02 mM MeOH
0.05 mM MeOH
0.1 mM MeOH
1 mM MeOH
GE-M14
GE-M24
GE-M4
MaE-M17
MiE-M1
RVI-57*
1
2
3
abundance (cells ml-1)
10⁸
10⁴
10⁰
0
25
50
days
'Ca. Methylopumilus universalis' - Czech lakes
no MeOH
0.02 mM MeOH
0.05 mM MeOH
0.1 mM MeOH
1 mM MeOH
MH-M4
MH-M5
RIV-30
RIV-50
abundance (cells ml-1)
days

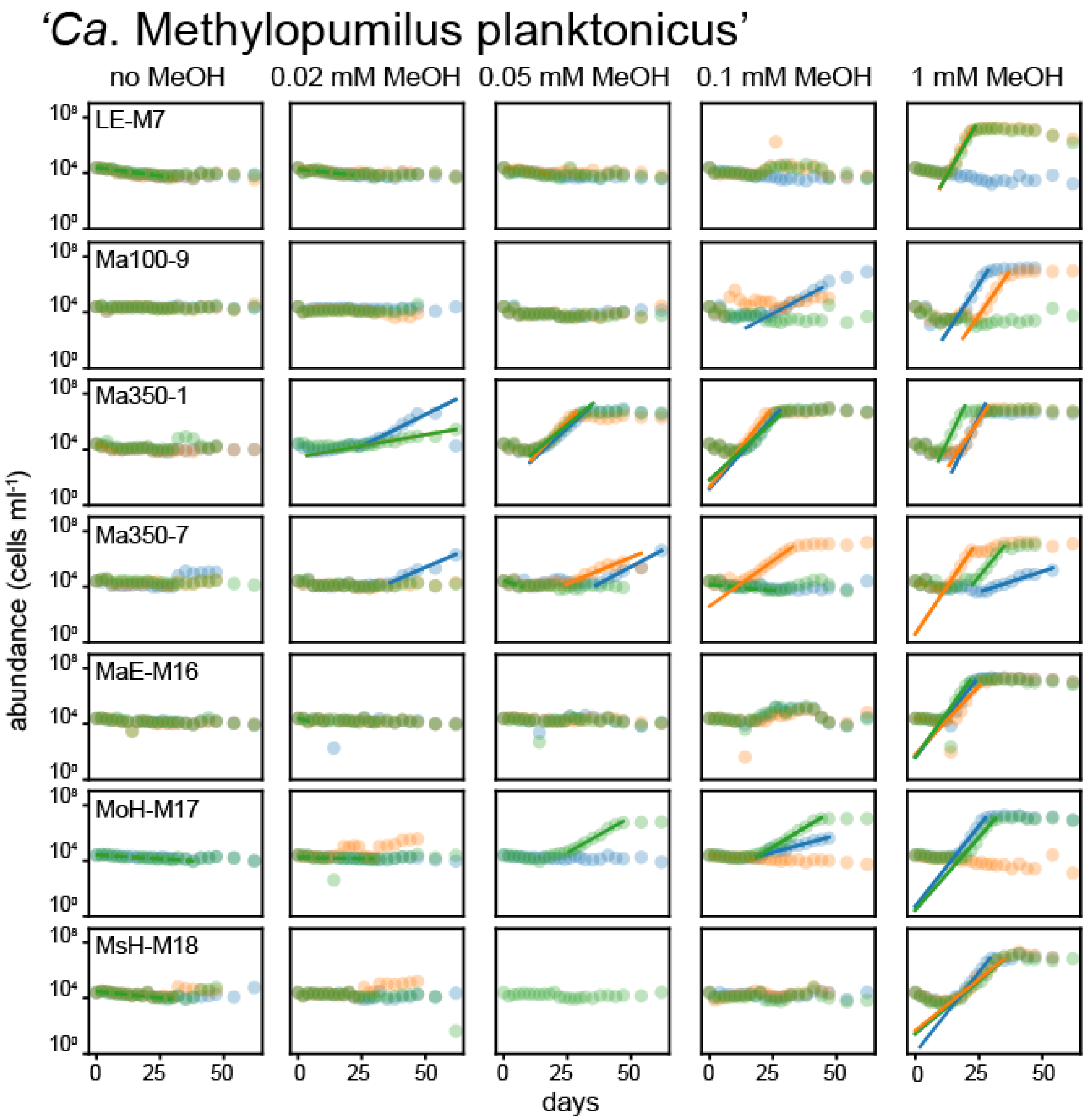

Figure S10: Individual growth curves of 10 '*Ca.* Methylopumilus universalis' and 7 '*Ca.* Methylopumilus planktonicus' isolates in triplicates under increasing methanol concentrations. Individual plots at 0, 0.02, 0.05, 0.1, and 1 mM methanol are shown, while 0.00 and 0.001 mM methanol are not displayed due to redundancy (no recorded growth). Specific growth rates shown in Fig. 4 were calculated for each strain based on fitted lines. *: RVI-57 was included in the non-Czech group as it grouped with the non-Czech sequences in the OstA tree (Fig. 4).
